# Dehydration induced transcriptomic responses in two Tibetan hulless barley (*Hordeum vulgare* var. *nudum*) accessions distinguished by drought tolerance

**DOI:** 10.1186/s12864-017-4152-1

**Published:** 2017-10-11

**Authors:** Junjun Liang, Xin Chen, Guangbing Deng, Zhifen Pan, Haili Zhang, Qiao Li, Kaijun Yang, Hai Long, Maoqun Yu

**Affiliations:** 1 Chengdu Institute of Biology, Chinese Academy of Sciences, Chengdu, 610041 People’s Republic of China; 2 CAS Key Laboratory of Mountain Ecological Restoration and Bioresource Utilization & Ecological Restoration and Biodiversity Conservation Key Laboratory of Sichuan Province, Chengdu Institute of Biology, Chinese Academy of Sciences, Chengdu, 610041 People’s Republic of China; 3 Center Laboratory Department, The General Hospital of Chengdu Army, Chengdu, 610083 People’s Republic of China; 4 Ganzi Tibetan Autonomous Prefecture Institute of Agricultural Science, Kangding, 626000 People’s Republic of China

**Keywords:** Tibetan hulless barley (*Hordeum vulgare* Var. *nudum*), Drought tolerance, Dehydration stress, RNA-sequencing, Transcriptome

## Abstract

**Background:**

The harsh environment on the Qinghai-Tibetan Plateau gives Tibetan hulless barley (*Hordeum vulgare* var. *nudum*) great ability to resist adversities such as drought, salinity, and low temperature, and makes it a good subject for the analysis of drought tolerance mechanism. To elucidate the specific gene networks and pathways that contribute to its drought tolerance, and for identifying new candidate genes for breeding purposes, we performed a transcriptomic analysis using two accessions of Tibetan hulless barley, namely Z772 (drought-tolerant) and Z013 (drought-sensitive).

**Results:**

There were more up-regulated genes of Z772 than Z013 under both mild (5439-VS-2604) and severe (7203-VS-3359) dehydration treatments. Under mild dehydration stress, the pathways exclusively enriched in drought-tolerance genotype Z772 included Protein processing in endoplasmic reticulum, tricarboxylic acid (TCA) cycle, Wax biosynthesis, and Spliceosome. Under severe dehydration stress, the pathways that were mainly enriched in Z772 included Carbon fixation in photosynthetic organisms, Pyruvate metabolism, Porphyrin and chlorophyll metabolism. The main differentially expressed genes (DEGs) in response to dehydration stress and genes whose expression was different between tolerant and sensitive genotypes were presented in this study, respectively. The candidate genes for drought tolerance were selected based on their expression patterns.

**Conclusions:**

The RNA-Seq data obtained in this study provided an initial overview on global gene expression patterns and networks that related to dehydration shock in Tibetan hulless barley. Furthermore, these data provided pathways and a targeted set of candidate genes that might be essential for deep analyzing the molecular mechanisms of plant tolerance to drought stress.

**Electronic supplementary material:**

The online version of this article (10.1186/s12864-017-4152-1) contains supplementary material, which is available to authorized users.

## Background

Drought is a major adversity that impacts plant growth, development, and productivity, and is a leading threat to the global food supply [1]. For survival, plants have evolved a complex mechanism of drought tolerance, which involves diverse gene expression patterns and as complex signaling pathways [2]. Understanding the mechanism of drought tolerance can help in improving the crop productivity [3]. Many drought-inducible genes with varying roles have been identified in *Arabidopsis*, *Triticum* species, and other species [1, 4–8]. Although much has been learnt from previous studies, our understanding of the response of plants to drought stress remains incomplete.

Barley (*Hordeum vulgare* L., 2n = 2× = 14) is the fourth most abundant cereal in the world (http://faostat.fao.org). Compared to its close relative, wheat, barley has a relatively small genome of 5.1 gigabases (Gbs) [9], and is more tolerant to drought [10]. Therefore, it can be used as a good model for the analysis of drought tolerance mechanism [10]. China is one of the places where barley originated. It produces large quantities of hulless barley (approximately 77% total reserves of the world), which plays an important part in the Tibetan life [11, 12]. The harsh environment on the Qinghai-Tibetan Plateau gives Tibetan hulless barley (*Hordeum vulgare* var. *nudum*) great ability to resist adversities such as drought, salinity, and low temperature, and it can, thus, serve as a good source for the breeding of drought-resistance alleles [13]. Identification of drought tolerance related genes in Tibetan hulless barley will enrich our knowledge of drought tolerance mechanisms, and might help in improving or stabilizing the crop yield in dry areas worldwide.

To understand the complex nature of drought tolerance, instead of looking at its individual components, a plant must be viewed as a complete system [14]. Transcriptomic analysis is an effective approach to identify drought stress related genes, pathways, and processes. The studies on molecular mechanisms of drought tolerance using transcriptomic analysis have been reported extensively in many plants [15–20], including wheat [21], wild emmer wheat [22, 23] and wild barley [24, 25]; however, little is known about the Tibetan hulless barley. Recently, Zeng et al. demonstrated changes in the gene expression patterns of well-watered, water deficit, and final water recovery stages in hulless barley. For constructing cDNA library, they evaluated the drought stress level of their samples by scoring the relative soil moisture content (RSMC) which was found to be 33.4%, 27.5%, 21.1%, 15.5%, 9.8%, and 4.8%, indicating that their drought stress treatment was slow and emulated the field conditions [26]. It was reported that the transcriptomic responses can be greatly affected by the rate of stress imposition; fast dehydrated (~6 h) and gradually dehydrated (~7 d) barley were demonstrated to have only 10% of the stress-responsive transcripts in common [27]. Thus, we still lack the information on transcriptomic changes under rapid dehydration stress in hulless barley.

Drought has a large influence on plant growth during germination, vegetative and reproductive stages [28]. The effects of terminal drought stress have been extensively studied in barley while the effects of drought during the juvenile stages were less well documented [29]. Sown in March every year, Tibetan hulless barley is usually affected by drought and low temperature at its seedling stage when the weather is cold and dry [29]. It is reported that when imposed during the early developmental stages, drought severely influences the development and final yield of barley [30]. Thus, determining the transcriptomic changes at the juvenile stages will provide useful data for enhancing our understanding of drought tolerance in hulless barley.

To elucidate specific gene networks and pathways that contribute to the tolerance of hulless barley to dehydration stress, in this study, we performed a transcriptomic analysis on the seedlings of two contrasting Tibetan hulless barley accessions, Z772 (drought-tolerant) and Z013 (drought-sensitive), using the Illumina HiSeqTM 2000 platform. The questions that we addressed were as follows: i) Which genes or pathways exhibit the most important differences between normal condition and dehydration stress? ii) Are there any molecular differences between the two contrasting genotypes during dehydration stress? Overall, we identified important genes and pathways related to dehydration stress in Tibetan hulless barley, which would provide practical knowledge for further expounding the specific mechanism of drought tolerance.

## Results and discussion

### Phenotypic responses to drought stress

**Fig. 1:**
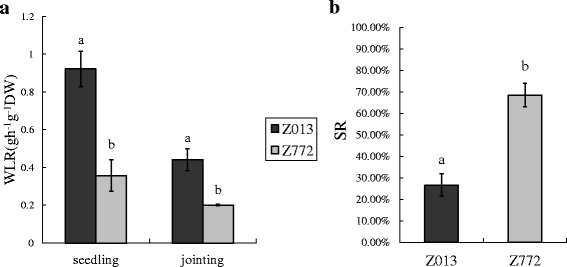
Water loss rate (WLR) and survival rate (SR) of Z772 and Z013. **a** The WLR of Z772 and Z013 in seedling and jointing stage, data was shown as the means ± S.D. **b** The SR of Z772 and Z013 in seedling stage, data represented the average of five experiments, were shown as the means ± S.E. (*n* = 5). The markers a and b on the top of each bar indicated that the means were significantly different at *P* = 0.05 as determined by the least significant difference (LSD) test using Duncan’s test (SPSS package, version 16.0)

Among the 48 Tibetan hulless barley accessions that were evaluated previously, Z772 and Z013 were identified as the most tolerant and sensitive, respectively [31]. To verify their drought tolerance, we tested the water loss rate (WLR) of the detached leaves and the survival rate (SR) under long-term drought stress. The results showed that the WLR of Z013 was significantly higher than that of Z772 at both seedling and jointing stages (Fig. 1a). The SR test also showed that compared to Z013, more Z772 plants survived after exposure to drought stress (Fig. 1b).

### RNA-Seq and transcriptome assembly

To obtain transcriptomic profiling of Tibetan hulless barley during water-deficit stress, total RNA from leaf samples of two contrasting accessions, Z772 and Z013, under water-deficit treatment of 0, 1 and 5 h were used to generate six independent libraries. The libraries prepared from samples of Z772 collected at 0, 1, and 5 h after dehydration were named as libraries A, B, and C whereas those prepared from the samples of Z013 were named as D, E, and F, respectively.

These libraries were sequenced using Illumina HiSeqTM 2000 platform, which generated more than 10 million 50 bp clean reads for each library. The results indicated that 78.20% (8,194,748)–81.90% (11,163,441) of these reads can be mapped to Tibetan hulless barley genome (Table 1).

**Table 1:** Summary of mapping result

Sample ID	Total Clean Reads	Total Reads Mapped on Hulless Barley Genome
A	14,027,376 (100.00%)	11,163,441 (79.58%)
B	10,892,772 (100.00%)	8,860,959 (81.35%)
C	10,478,758 (100.00%)	8,194,748 (78.20%)
D	11,250,915 (100.00%)	9,214,076 (81.90%)
E	10,479,575 (100.00%)	8,439,993 (80.54%)
F	11,535,804 (100.00%)	9,185,349 (79.62%)

A: Z772 0 h, B: Z772 1 h, C: Z772 5 h, D: Z013 0 h, E: Z013 1 h, F: Z013 5 h

### qRT-PCR validation

**Fig. 2:**
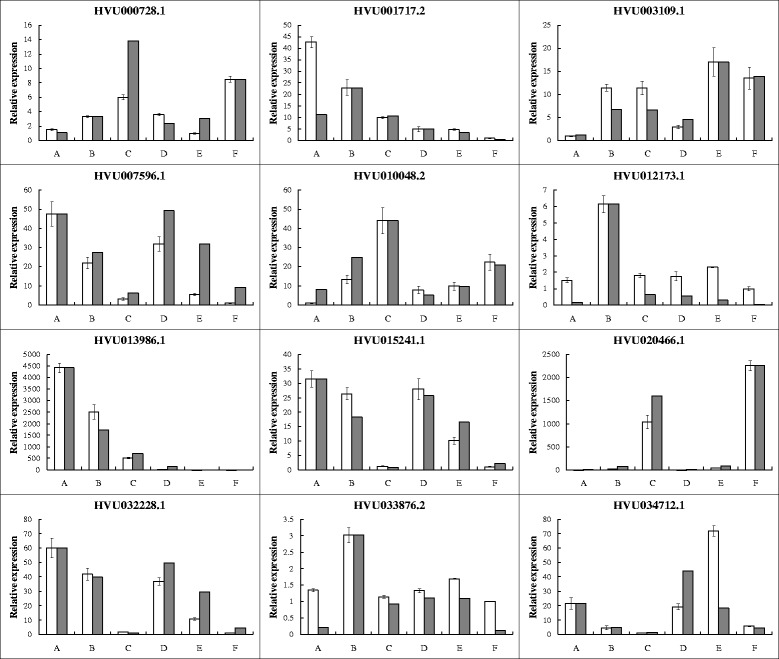
Quantitative real-time PCR (qRT-PCR) validation of 12 differentially expressed genes. Accumulations of 12 genes were analyzed by qRT-PCR using *EF1α* as internal control under dehydration stress for 0, 1, and 5 h. Data was shown as means ± S.D. (*n* = 4). White and gray bars represented for qRT-PCR results and RNA-Seq data, respectively. Gene-specific primers used for real-time PCR were listed in Additional file 1

To validate the accuracy and repeatability of our RNA-Seq data, 12 genes were selected for quantitative real-time polymerase chain reaction (qRT-PCR) analysis (Additional file 1). As shown in Fig. 2, the results of qRT-PCR indicated that most of these genes had expression patterns that agreed with the RNA-Seq data, testifying the reliability of our data. The results also indicated that a few genes showed different expression change at one or two time points between the two methods. In fact, this discrepancy was observed in other studies as well [32–34]; however, the reasons for this discrepancy remain unclear. One reason may be the use of elongation factor 1α (*EF1α*) as the reference gene. Although it is the best among the traditional reference genes, such as *glyceraldehyde-3-phosphate dehydrogenase* (*GAPDH*), *β-Actin*, *β-Tubulin*, and *ubiquinone* (*UBQ*) (Additional file 2), its expression pattern is not completely invariable during dehydration stress.

### Profile of RNA expression in tolerant and sensitive accessions

**Fig. 3:**
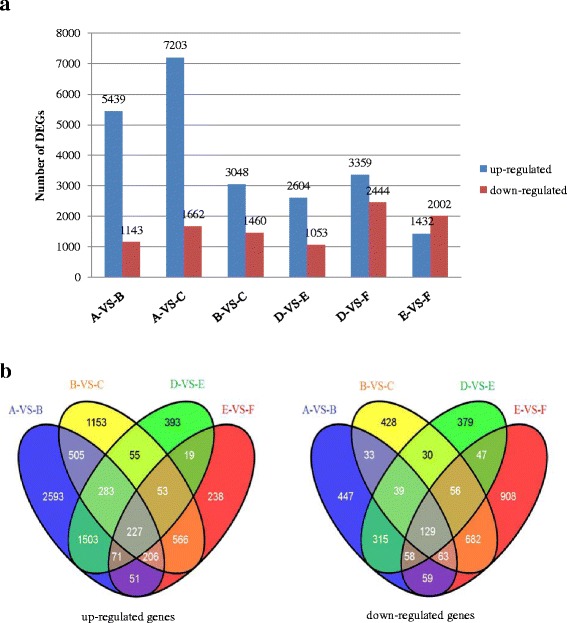
Overview of differentially expressed genes (DEGs). **a** Pairwise comparison of DEGs. In a pairwise comparison (denote as A-VS-B for example), the former one (A) was considered as the control, and the latter one (B) was considered as the treatment, the same below. **b** Venn diagrams showing the number of transcripts which overlaps among DEGs in Z772 and Z013**.** The diagram at left showed up-regulated genes at 0, 1 and 5 h after dehydration stress. The diagram at right showed down-regulated genes. Only transcripts with a change of >2 fold were included

An overview of the differentially expressed genes (DEGs) is provided in Fig. 3a. The number of up-regulated genes in Z772 was much more than in Z013 after 1 h (5439-VS-2604) and 5 h (7203-VS-3359) of dehydration stress, whereas differences in the number of down-regulated genes were less obvious (1143-VS-1053 at 1 h, 1662-VS-2444 at 5 h). These results may suggest that Z772 can actively respond to drought stress by enhancing the expression of more drought related genes.

The DEGs identified in the four comparisons (A-VS-B, B-VS-C, D-VS-E and E-VS-F) were analyzed using a Venn diagram (Fig. 3b). The common regions of A-VS-B and B-VS-C in the section of up-regulated genes contained 1221 genes, which represented only 16.80% of the total number of 7266 up-regulated genes in Z772. The common regions of D-VS-E and E-VS-F in the section of up-regulated genes also contained only a small proportion (370 unigenes, 10.10%) in Z013. These results indicated that a large number of genes responded to drought stress in a stage-specific manner, and that the gene expression patterns under mild and severe dehydration stress were quite different.

### GO and KEGG enrichment

**Fig. 4:**
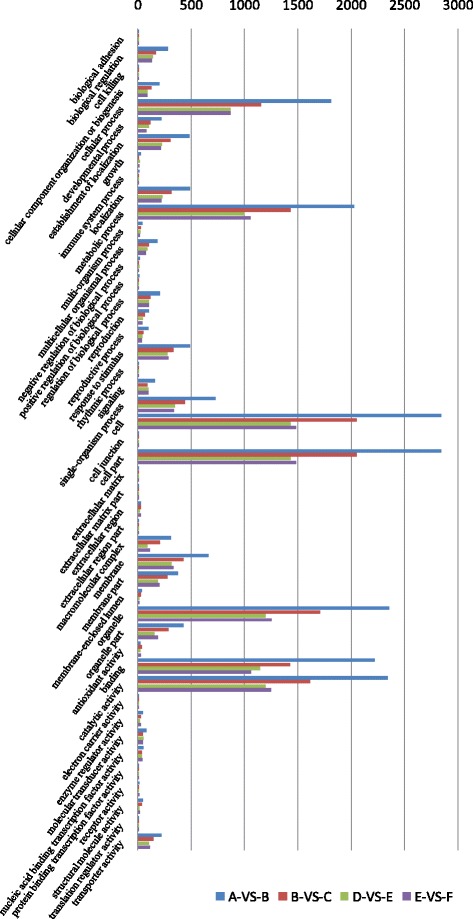
Gene ontology (GO) functional classification analysis of dehydration stress related DEGs based on RNA-Seq data. GO functional classification analysis of DEGs in A-VS-B, B-VS-C, D-VS-E, E-VS-F were represented using blue, red, green and purple bars respectively

Gene ontology (GO) functional classification analysis was carried out to categorize the functions of DEGs during dehydration stress. The DEGs could be classified into three main ontologies, namely Molecular function, Biological process, and Cellular component, which included 22, 13, and 12 functional groups, respectively (Fig. 4).

Similar distributions were found in both Z772 and Z013. In the Biological process category, DEGs were basically enriched in cellular process, and metabolic process. As in the Cellular component category, DEGs were primarily enriched in cell, cell part, and organelle. With regard to the Molecular function category, the most enriched GO terms were catalytic activity and binding (Fig. 4). Remarkably, DEGs of Z772 under mild dehydration stress (after 1 h dehydration treatment) were far more than those under severe dehydration stress (after 5 h dehydration treatment) in these GO terms, but DEGs of Z013 between mild and severe dehydration stress were not notable.

**Fig. 5:**
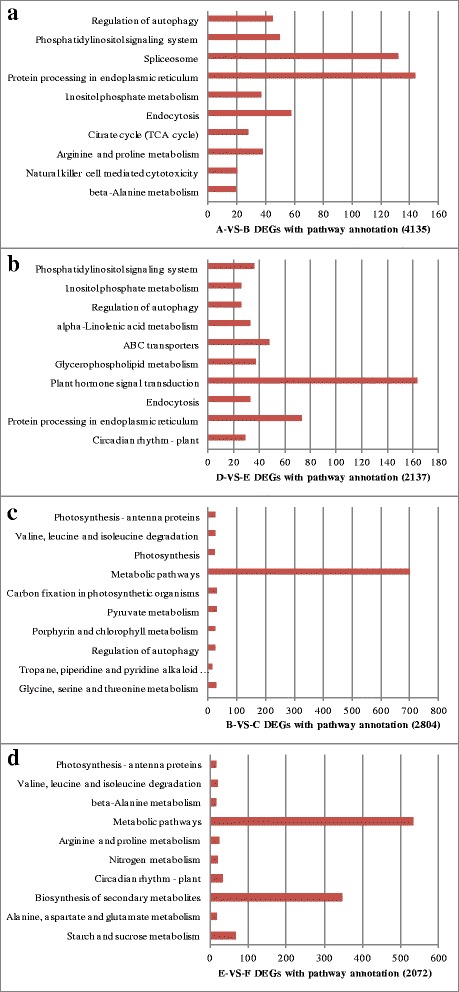
KEGG pathway annotations of DEGs in different comparative pairs. **a**, **b**, **c** and **d** represented for A-VS-B, D-VS-E, B-VS-C and E-VS-F, respectively

To further gain insights into the biological functions and interactions of the DEGs, the Kyoto Encyclopedia of Genes and Genomes (KEGG) Pathway enrichment analysis was carried (Fig. 5). The primary pathways affected in both Z772 and Z013 by mild dehydration stress included Phosphatidylinositol signaling system; Regulation of autophagy; Inositol phosphate metabolism; Endocytosis. Pathways exclusively enriched in Z772 included Protein processing in endoplasmic reticulum (which was discussed in detail in the following section); tricarboxylic acid (TCA) cycle; Wax biosynthesis; Spliceosome; Natural killer cell mediated cytotoxicity (Additional files 3, 4, 5, 6). Pathways exclusively enriched in Z013included ATP-binding cassette transporters (ABC transporters); Alpha-Linolenic acid metabolism; Plant hormone signal transduction; Circadian rhythm-plant.

Under severe dehydration stress, pathways enriched in both accessions mainly included Photosynthesis-antenna proteins; Valine, leucine and isoleucine degradation; Metabolic pathways. Pathways mainly enriched in Z772 included Carbon fixation in photosynthetic organisms; Pyruvate metabolism; Porphyrin and chlorophyll metabolism; Regulation of autophagy; Tropane, piperidine and pyridine alkaloid biosynthesis; Glycine, serine and threonine metabolism. Pathways mainly enriched in Z013 included beta-Alanine metabolism; Starch and sucrose metabolism.

### KEGG pathway visualization of “protein processing in endoplasmic reticulum”

**Fig. 6:**
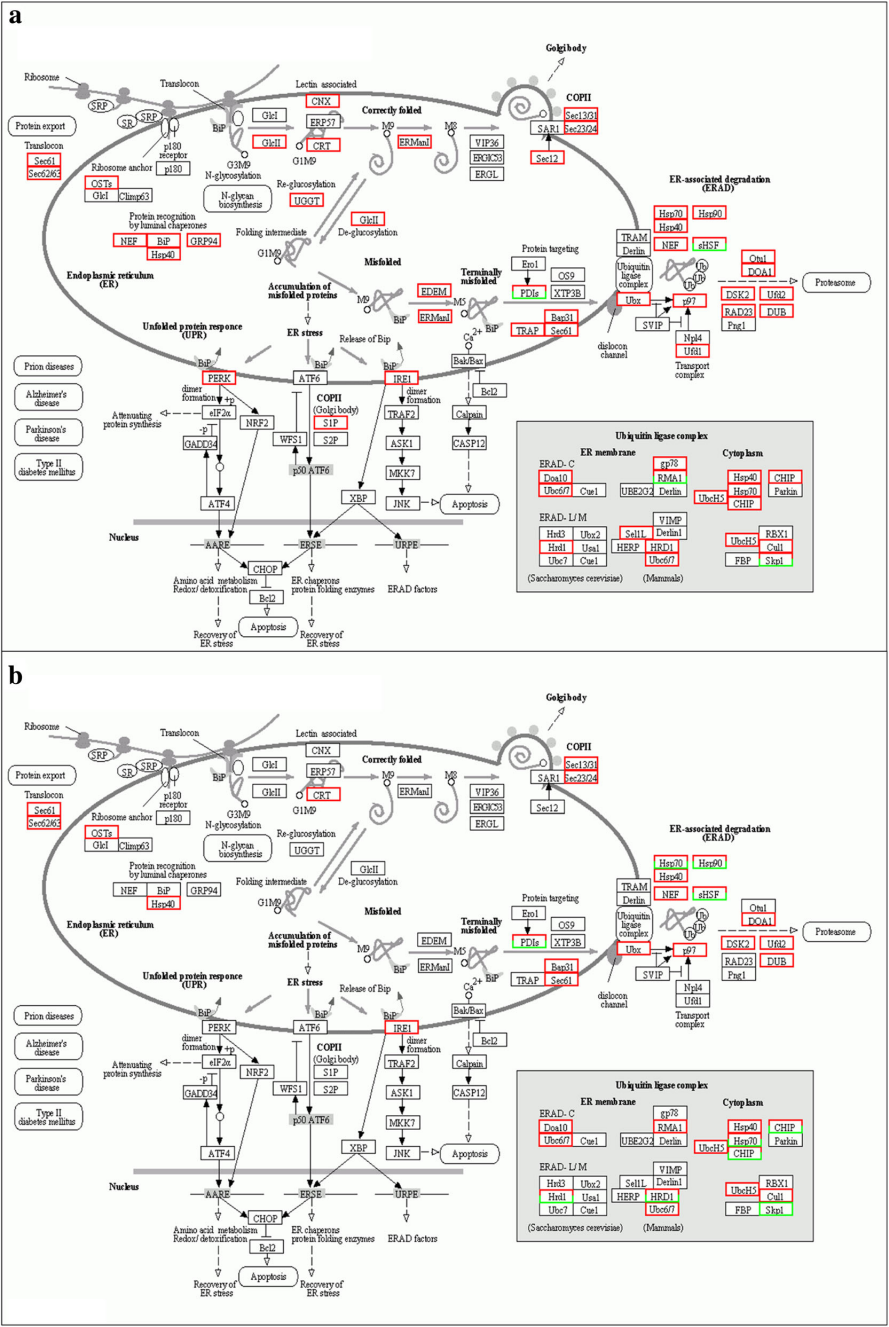
KEGG pathway visualization of Protein processing in endoplasmic reticulum associated DEGs. **a** A-VS-B. **b** D-VS-E. Genes which were coded red were up-regulated and green were down-regulated

Unfolding or misfolding of proteins is the greatest risk during drought stress [35]. Thus, protein processing in endoplasmic reticulum is a very important pathway under dehydration stress. The key genes in this pathway were discovered and compared between A-VS-B and D-VS-E (Fig. 6). There were 144 DEGs in A-VS-B, with six of them down-regulated; there were 73 DEGs in D-VS-E, with eight of them down-regulated (Fig. 6). These data indicated that most of the genes related to protein processing were up-regulated under drought stress to promote the efficiency of protein processing for stress response.

In addition, the number of genes which were up-regulated was considerably higher in Z772 than in Z013. The prominent differences between A-VS-B and D-VS-E were protein recognition by luminal chaperones, deglucosylation, and reglucosylation. Other differences between A-VS-B and D-VS-E were enriched in eukaryotic translation initiation factor, calnexin, mannosyl-oligosaccharide alpha-1, 2-mannosidase, and heat shock proteins (hsps), such as hsp 40, 70, and 90. These differences suggested that Z772 has a mechanism to increase the accuracy of protein folding, and could, thus, facilitate its processing of drought related proteins better.

### The main up and down-regulated transcripts

The main DEGs in A-VS-B, D-VS-E, B-VS-C and E-VS-F were shown in Table 2 and Additional files 7, 8, and 9, respectively. The most highly elevated genes under dehydration stress in both Z772 and Z013 were those encoding dehydrins, which are hydrophilic and reliably thermostable, produced in response to high temperature and osmotic stress [35–40]. Most dehydrins can be classified into Group II Late Embryogenesis Abundant (LEA) family, which function in stabilizing labile enzymes, binding water, and protecting macromolecular structures under abiotic stress. Other members of LEAs were also found as highly elevated genes in this study, including LEA1, HVA22, and Dhn8. The up-regulated expression pattern of LEA genes under drought stress was not only restricted in leaves, but also been reported in lemma, palea, and awn in barley [35].

**Table 2:** The annotated 72 genes in top 100 genes differentially expressed in response to mild dehydration stress compared to unstressed condition (based on log2 Ratio of FDR) in Z772

GeneID	Annotation	log2 Ratio	Regulation
HVU035383.1	dehydrin	5.74	Up
HVU003154.1	CTP synthase	5.59	Up
HVU004518.1	copper chaperone	5.51	Up
HVU006762.1	LRR receptor-like serine/threonine-protein kinase EFR	5.24	Up
HVU021557.1	trehalose 6-phosphate synthase/phosphatase	5.16	Up
HVU009064.1	9-cis-epoxycarotenoid dioxygenase	4.76	Up
HVU037206.1	ATP-dependent Clp protease ATP-binding subunit ClpC	4.57	Up
HVU002314.1	spermidine synthase	4.57	Up
HVU005463.1	F-box and leucine-rich repeat protein 2/20	4.47	Up
HVU021987.1	solute carrier family 36	4.45	Up
HVU032011.1	U4/U6 small nuclear ribonucleoprotein PRP3	4.32	Up
HVU006047.1	homeobox-leucine zipper protein	4.30	Up
HVU007810.1	hydroperoxide dehydratase	4.21	Up
HVU033324.1	gelsolin	4.18	Up
HVU018901.1	DNA-directed RNA polymerase III subunit RPC2	4.14	Up
HVU037126.1	DNA topoisomerase 2-associated protein PAT1	4.00	Up
HVU029765.1	MFS transporter, OCT family, solute carrier family 22	3.82	Up
HVU014205.1	protein phosphatase 2C	3.70	Up
HVU037033.1	beta-fructofuranosidase	3.69	Up
HVU027000.1	translation initiation factor 5B	3.59	Up
HVU002284.1	ATP-dependent Clp protease ATP-binding subunit ClpB	3.53	Up
HVU015814.2	farnesyl-diphosphate farnesyltransferase	3.42	Up
HVU011974.1	DNA-directed RNA polymerase III subunit RPC2	3.29	Up
HVU036846.1	arabidopsis histidine kinase 2/3/4 (cytokinin receptor)	3.21	Up
HVU005864.1	respiratory burst oxidase	3.12	Up
HVU007764.1	DnaJ homolog subfamily A member 2	3.09	Up
HVU037797.1	beta-glucosidase	3.06	Up
HVU017010.1	cellulose synthase A	3.00	Up
HVU025623.1	glutamate synthase (NADPH/NADH)	3.00	Up
HVU005697.1	protein phosphatase 2C	2.94	Up
HVU031846.1	non-specific polyamine oxidase	2.94	Up
HVU004727.1	trehalose 6-phosphate synthase/phosphatase	2.89	Up
HVU003036.1	ubiquitin C	2.86	Up
HVU003109.1	glutathione S-transferase	2.49	Up
HVU004122.1	beta-amylase	2.49	Up
HVU002272.1	stress-induced transcription factor SNAC1	2.38	Up
HVU038692.1	solute carrier family 15	2.37	Up
HVU007121.1	EREBP-like factor	2.36	Up
HVU015589.1	heat shock protein 90 kDa beta	2.30	Up
HVU026868.1	ATP-dependent Clp protease ATP-binding subunit ClpC	2.23	Up
HVU007682.1	signal recognition particle receptor subunit alpha	2.10	Up
HVU015402.1	beta-fructofuranosidase	2.09	Up
HVU004540.2	cytochrome P450, family 71, subfamily D, polypeptide 9	2.02	Up
HVU028755.1	hydroperoxide dehydratase	1.98	Up
HVU034249.1	ubiquitin-conjugating enzyme E2 D/E	1.85	Up
HVU025026.1	4-hydroxy-3-methylbut-2-enyl diphosphate reductase	1.83	Up
HVU018834.1	glutamate--glyoxylate aminotransferase	1.78	Up
HVU015735.2	ribulose-phosphate 3-epimerase	1.71	Up
HVU031949.2	glutamate synthase (ferredoxin)	1.66	Up
HVU021798.1	sucrose synthase	1.62	Up
HVU005506.1	solute carrier family 35, member E1	1.31	Up
HVU002709.1	phosphoribulokinase	1.27	Up
HVU002466.1	auxin responsive GH3 gene family	1.27	Up
HVU029346.1	glycine hydroxymethyltransferase	1.23	Up
HVU036753.1	paf93	1.19	Up
HVU016880.1	fructose-bisphosphate aldolase, class I	−2.52	Down
HVU007595.1	ferredoxin	−2.53	Down
HVU011147.3	two-component response regulator ARR-B family	−2.55	Down
HVU023945.2	cyanohydrin beta-glucosyltransferase	−2.55	Down
HVU024713.1	pyridoxine biosynthesis protein	−2.64	Down
HVU020110.1	gibberellin receptor GID1	−2.76	Down
HVU038925.4	cytochrome P450, family 71, subfamily Z, polypeptide 6 (ent-isokaurene C2-hydroxylase)	−3.01	Down
HVU014053.1	histone H1/5	−3.09	Down
HVU036050.2	aquaporin PIP	−3.37	Down
HVU021282.1	lycopene beta-cyclase	−3.44	Down
HVU021893.1	cyanohydrin beta-glucosyltransferase	−3.6	Down
HVU011440.1	chalcone synthase	−3.85	Down
HVU024685.1	threonine-protein kinase SRPK3	−3.92	Down
HVU008611.1	hydroquinone glucosyltransferase	−3.98	Down
HVU011027.1	phenylalanine ammonia-lyase	−4.08	Down
HVU014010.1	chalcone isomerase	−4.35	Down
HVU015232.1	peroxidase	−4.79	Down

Other highly elevated functional genes included solute carrier (SLC) family 36, an amino acids transport proteins [41]; trehalose 6-phosphate synthase/phosphatase, which catalyzes the synthesis of trehalose (a nonspecific protective agent for biomacromolecules). It was reported the trehalose pathway has an association with abiotic stress tolerance [42]; Δ1-pyrroline-5-carboxylate synthase (P5CS), a key enzyme of glutamate pathway in the synthesis of proline, which is believed to play critical roles in promoting drought tolerance [43]. However, the glutamate pathway is not the only way for the biosynthesis of Pro. It was reported that the expression of P5CS gene did not changed in the spike of barley under drought [35], which suggested the biosynthesis of Pro preferred to via the Orn pathway in barley spike during the process of dehydration; wheat cold-responsive (WCOR)413 and 615, their accumulation under dehydration suggested that some of the freezing tolerance genes might also participate in drought tolerance; asparagine synthase, which is up-regulated by salt, osmotic, and abscisic acid (ABA) treatment in wheat [44] and is believed to enhance detoxification in drought-tolerant cotton varieties [45]; hsps can bind to unfolded proteins, stabilize the protein tertiary structure and block intermolecular interactions. One hsp 20, one hsp 70 and two hsp 90 genes were up-regulated in leaves of both Z772 and Z013. The up-regulated patterns of other *hsp* genes were also reported previously in drought-stressed lemma, palea, and awn in barley [35].

The regulatory genes with highly elevated expression included protein phosphatase 2C (PP2C), a family of protein phosphatases, which are key players in plant signal transduction processes [46, 47]; Ca^2+^-transporting ATPase, which serves to maintain low concentrations of Ca^2+^ for proper cell signal transduction; WRKY transcription factor, a class of DNA-binding proteins; NAC transcription factor, which play important roles in plant development and stress responses [48–50]; allene oxide synthase (hydroperoxide dehydratase), catalyzes the first step in the biosynthesis of jasmonic acid, which functions in regulating plant responses to biotic and abiotic stresses [51]. Most of these regulatory genes showed an initial increased pattern under mild dehydration stress and then a decreased or unchanged pattern under severe dehydration stress.

The genes whose expression was most highly reduced in both Z772 and Z013 were a group of plant aquaporins, the aquaporin PIP (plasma membrane intrinsic) protein, which regulate water conductance of the plasma membrane [52–55]. Among the 12 PIPs which were detected in this study, only one showed an up-regulated pattern. Twenty of the 21 genes encoding the light-harvesting complex II chlorophyll a/b binding protein 1 were found to be drastically down-regulated. Considering that these genes are involved in photosystem (PS) I and II, their suppression indicates that photosynthesis might be repressed during dehydration in leaves. The down-regulation of genes involved in photosynthesis was also reported in drought-stressed spike organs in barley previously [35]. Other highly reduced genes included fructose-bisphosphate aldolase (class I), ferredoxin, chalcone synthase and chalcone isomerase, histone H1/5 and histone H4, hydroquinone glucosyltransferase, and lycopene beta-cyclase.

### Identification of genes responding only in drought tolerant genotype

We analyzed the DEGs between tolerant and sensitive genotypes and the results indicated that there were much more DEGs which expressed uniquely in the tolerant genotype Z772 (5159 unigenes) than in the sensitive genotype Z013 (1984 unigenes). The genes whose expression was highly elevated only in Z772 included Phospholipase C (PLC), a class of enzymes that cleave the phospholipid phosphatidylinositol 4,5-bisphosphate (PIP2) into diacylglycerol (DAG) and inositol 1,4,5-trisphosphate (IP3), participating in signal transduction; Squalene monooxygenase, which uses nicotinamide adenine dinucleotide phosphate (NADPH) and molecular oxygen to oxidize squalene to 2,3-oxidosqualene (squalene epoxide); thiamine biosynthesis protein ThiC, which is involved in the synthesis of thiamine (vitamin B1); trafficking protein particle complex subunit 6B, which might play a role in vesicular transport from endoplasmic reticulum to Golgi.

The genes with highly reduced expression only in Z772 included light-harvesting complex I chlorophyll a/b binding protein 3 and light-harvesting complex II chlorophyll a/b binding protein 2 and 3, their suppression suggested that Z772 might be better than Z013 in repressing its photosynthesis during dehydration. Other highly reduced genes only in Z772 included ribulose-bisphosphate carboxylase small chain, a component of ribulose-1, 5-bisphosphate carboxylase/oxygenase (RubisCO), which is involved in the first major step of carbon fixation; fructose-1,6-bisphosphatase I, which converts fructose-1,6-bisphosphate to fructose 6-phosphate (also involved in carbon fixation). Their suppression suggested that Z772 represses its carbon fixation during dehydration.

### Candidate genes for enhancing drought tolerance

**Fig. 7:**
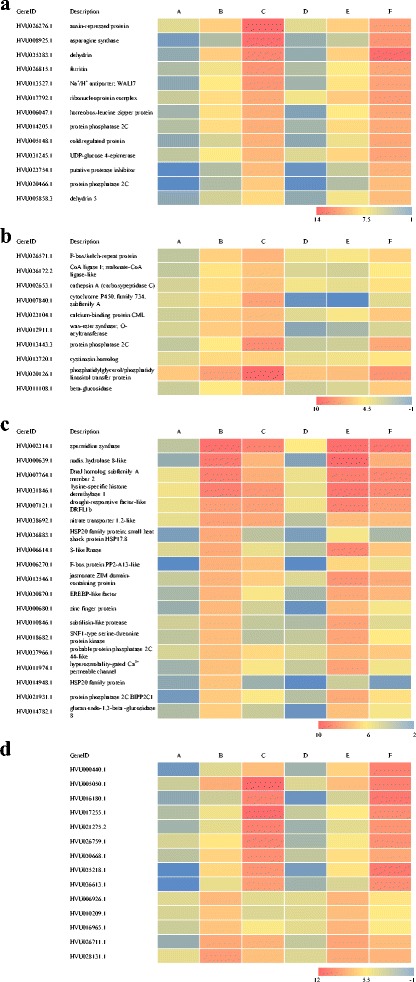
Heat map of candidate genes. **a** 13 dehydration induced candidate genes in both Z772 and Z013. **b** 10 dehydration induced candidate genes whose expression was significant higher in Z772 than in Z013. **c** 19 dehydration induced candidate genes which was highly up-regulated at 1 h but down-regulated or unchanged at 5 h. **d** 14 dehydration induced candidate genes without any annotation

Based on their expression patterns, 56 drought-induced genes were selected for further study. These candidate genes were divided into four groups. Firstly, we focused on the genes that showed a continued up-regulated pattern during dehydration stress in both Z772 and Z013 (Fig. 7a). These genes included auxin-repressed protein, asparagine synthetase, dehydrins, ferritin, and Na^+^/H^+^ antiporter, among others. Secondly, we considered the genes whose expression showed a continued up-regulated pattern but their expression was higher in Z772 compared to that in Z013 at least at 1 h of dehydration stress (Fig. 7b). These genes included F-box/kelch-repeat protein, Malate-CoA ligase, cathepsin A, cytochrome P450, calcium-binding protein CML, wax-ester synthase, and PP2C, among others. We also focused on those genes, which were highly up-regulated at 1 h but were down-regulated or remained unchanged at 5 h (Fig. 7c). These genes included spermidine synthase, nudix hydrolase 8, chaperone protein dnaJ (also known as hsp40), polyamine oxidase, and APETALA2/ethylene-responsive element binding protein (AP2/EREBP)-like transcription factor, among others. A total of 14 unannotated DEGs that met our above-mentioned requirements were also noticeable (Fig. 7d). The gene IDs, annotation, and reads per kilobases per million reads (RPKM)-values for the suggested candidate genes were shown in Fig. 7a, b, c, and d. A detailed description of these candidate genes was shown in Additional file 10.

## Conclusions

From what we know, it is the first study to measure the transcriptomic changes under detached dehydration stress in Tibetan hulless barley using RNA-Seq. The results indicated that the transcriptional regulation in Z772 and Z013 under dehydration stress was quite different, especially under conditions of mild dehydration stress. The pathways of Protein processing in endoplasmic reticulum, TCA cycle, Wax biosynthesis, and Spliceosome were mainly enriched in Z772 compared to that in Z013, indicating that the dehydration tolerant Z772 has a stronger ability to regulate protein synthesis and energy metabolism under stress conditions compared to Z013. A total of 56 drought-tolerant candidate genes were identified by their expression patterns; these genes could be used for genetic engineering or for marker assisted selection to enhance drought tolerance in hulless barley as well as in other crops. Overall, our data identify the pathways and a targeted set of candidate genes that might be essential for an in-depth analysis of the molecular mechanisms for the tolerance of plants to drought stress.

## Methods

### Plant materials and growth conditions

Two Tibetan hulless barley accessions (Z772 and Z013) were used in this study. Their tolerance to drought was identified by Liang et al. in the previous study [31]. Half-strength Murashige and Skoog (MS) solid medium was used for seeds germination. After 3 days, they were transplanted into plastic pots (with a height of 5 cm and a diameter of 5 cm; one plant per pot). The pots have 100 g of potting mixture which consisted by local soil, nutrient soil, and vermiculite in the ratio of 4:1:1 by volume. Hulless barley seedlings were grown in a greenhouse with a temperature of 23 to 25 °C, a relative humidity of 50% to 70%, and a photoperiod of 16 h/8 h light/dark at Chengdu Institute of Biology, Chinese Academy of Sciences (Chengdu, Sichuan, China).

### Drought stress treatment

All seedlings were well watered before stress. At five leaf stage (25 days after sowing, DAS), the most recently expanded fifth leaves were cut and put on filter paper in dry dishes in a growth chamber. The chamber has a constant temperature of 23 to 25 °C and a relative humidity of 40% to 60%. Equal amounts of leaves from 10 individuals of the two identical accessions were collected and pooled, after leaving on filter papers for 0, 1 and 5 h, respectively. These six pools were quickly grinded with a mortar and pestle using liquid nitrogen, and then stored in −80 °C refrigerator.

### RNA extraction and cDNA library construction

Total RNA was extracted from each of the six resulting samples using Trizol reagent (Invitrogen) with Pre-mix and purified using the RNeasy Plant Mini kit (Qiagen). These samples were treated with DNase I to degrade DNA and chromatin. NanoDrop 1000 spectrophotometer and formaldehyde-agarose gel electrophoresis were adopted to confirm the integrity and quality of the total RNA. Poly(A) mRNA was isolated by beads with oligo(dT) and then interrupted to short fragments (about 200 bp) by fragmentation buffer. Taking these short fragments as templates, the first-strand cDNA was synthesized by using reverse transcriptase and random hexamer-primers. Then the second strand were synthesized with buffer, dNTPs, RNase H and DNA polymerase I were added to. The double strand cDNA was purified with magnetic beads. End reparation and 3′-end single nucleotide A (adenine) addition was then performed. Finally, sequencing adaptors were ligated to the fragments. The fragments were enriched by PCR amplification. During the QC (quality control) step, Agilent 2100 Bioanaylzer and ABI Step One Plus Real-Time PCR System were used to qualify and quantify of the sample library. The final library products were sequenced on the Illumina HiSeqTM 2000 according to the manufacturer’s recommendations (Illumina).

### Alignment with the barley genome

The RNA-Seq reads produced by the HiSeqTM 2000 were initially processed to clean reads. Reads with adaptor sequences, more than 10% unknown bases, and low quality sequences in which more than 50% of the quality values less than five were removed. Then clean reads were mapped to the Tibetan hulless barley genome using SOAP2 [56]. In the alignment, at most two mismatches were allowed.

### Screening of DEGs

RPKM method was adopted to calculate gene expression level [57], using the formula as follows:

$$ RPKM=\frac{10^6C}{NL/{10}^3} $$

RPKM is the expression level of gene A, N represents total number of reads that uniquely aligned to all genes, C represents number of reads that uniquely aligned to gene A, and L represents number of bases of gene A.

If a gene has more than one transcript, its expression level and coverage calculates using the longest one.

### Expression pattern analysis of DEGs

We initially screened differentially expressed genes among samples, referring to “The significance of digital gene expression profiles [58]”. Then GO and KEGG enrichment analysis were performed for these DEGs.

We use “false discovery rate (FDR) ≤ 0.001 and the absolute value of log2Ratio ≥ 1” as the threshold to judge the significance of gene expression difference and DEGs should have smaller FDR and bigger fold-change value.

GO functional enrichment: The results that generated from basic local alignment search tool (BLAST) (with parameters: -p blastx -e 1e-5 -m 7) sequences to the Nr nucleotide database maintained by National Center for Biotechnology Information (NCBI) were annotated to the terms of GO [59] by BLAST2GO [60] (default parameters). KEGG pathway enrichment: Annotating to the KEGG [61] database through BLAST (with parameters:-p blastx -e 1e-5 -m 8).

### qRT-PCR validation

To validate the results of RNA-Seq, 12 genes were selected as targets for quantitative real-time PCR analysis. The first-strand cDNA was synthesized using 5 μg RNA samples and M-MLV reverse transcriptase (TaKaRa). The cDNA product was diluted ten times, and 1 μL was used in a 20-μL PCR reaction.

The PCR amplification consisted of a preincubation at 95 °C for 5 min and 40 cycles each 15 s at 95 °C, 15 s at 60 °C, and 15 s at72°C. These reactions used the Chromo4 real-time PCR detector system (Bio-Rad, USA) and iQ SYBR green supermix (Bio-Rad). To normalize the cDNA templates, the housekeeping gene *EF1α* was co-amplified. All primers (Additional file 1) were synthesized by Invitrogen.

### Data analysis

Data analysis was done on a completely randomized design. WLR data were analyzed by using one-way analysis of variance (ANOVA) and the mean differences were analyzed using least significant difference (LSD) test by SPSS package (version 16.0).

## Additional files

Additional file 1: Primers sequences and their product size in qRT-PCR. (XLSX 11 kb)

Additional file 2: The expression pattern of five housekeeping genes. (*GAPDH*, *EF1α*, *β-Actin*, *β-Tubulin* and *UBQ*). (XLSX 11 kb)

Additional file 3: KEGG pathway visualization of TCA cycle. (PDF 53 kb)

Additional file 4: KEGG pathway visualization of Wax biosynthesis. (PDF 95 kb)

Additional file 5: KEGG pathway visualization of Spliceosome. (PDF 65 kb)

Additional file 6: KEGG pathway visualization of Natural killer cell mediated cytotoxicity. (PDF 101 kb)

Additional file 7: The annotated 80 genes in top 100 genes differentially expressed in response to light dehydration stress compared to unstressed control (based on log2 Ratio of FDR) in Z013. (XLSX 14 kb)

Additional file 8: The annotated 77 genes in top 100 genes differentially expressed in response to heavy dehydration stress compared to light dehydration stress (based on log2 Ratio of FDR) in Z772. (XLSX 13 kb)

Additional file 9: The annotated 71 genes in top 100 genes differentially expressed in response to heavy dehydration stress compared to light dehydration stress (based on log2 Ratio of FDR) in Z013. (XLSX 13 kb)

Additional file 10: Candidate genes to enhance drought tolerance. (DOCX 29 kb)

## Abbreviations

ABA: Abscisic acid
ABC transporters: ATP-binding cassette transporters
ANOVA: Analysis of variance
AP2/EREBP: APETALA2/ethylene-responsive element binding protein
BLAST: Basic local alignment search tool
Ct: Threshold cycle
DAG: Diacylglycerol
DAS: Days after sowing
DEGs: Differentially expressed genes
EF1α: Elongation factor 1α
FDR: False discovery rate
GAPDH: Glyceraldehyde-3-phosphate dehydrogenase
Gbs: Gigabases
GO: Gene ontology
HSPs: Heat shock proteins
IP3: Inositol 1, 4, 5-trisphosphate
KEGG: Kyoto encyclopedia of genes and genomes
LEA: Late embryogenesis abundant
LSD: Least significant difference
MS: Murashige and Skoog
NADPH: Nicotinamide adenine dinucleotide phosphate
NCBI: National center for biotechnology information
P5CS: Δ1-pyrroline-5-carboxylate synthase
PIP: Plasma membrane intrinsic
PIP2: Phospholipid phosphatidylinositol 4, 5-bisphosphate
PLC: Phospholipase C
PP2C: Protein phosphatase 2C
PS: Photosystem
QC: Quality control
qRT-PCR: Quantitative real-time polymerase chain reaction
RPKM: Reads per kilobases per million reads
RSMC: Relative soil moisture content
RubisCO: Ribulose-1, 5-bisphosphate carboxylase/oxygenase
SE: Standard error
SLC: Solute carrier
SR: Survival rate
SRA: Short read archive
TCA: Tricarboxylic acid
UBQ: Ubiquinone
WCOR: Wheat cold-responsive
WLR: Water loss rate
