# Supplementary material for: Dehydration induced transcriptomic responses in two Tibetan hulless barley (Hordeum vulgare var. nudum) accessions distinguished by drought tolerance
Source: BMC Genomics. 2017 Oct 11;18:775. doi: 10.1186/s12864-017-4152-1 (PMC5637072; doi:10.1186/s12864-017-4152-1)
Supplement: Supplementary file 3 — KEGG pathway visualization of TCA cycle. (PDF 53 kb) [file 12864_2017_4152_MOESM3_ESM.pdf]

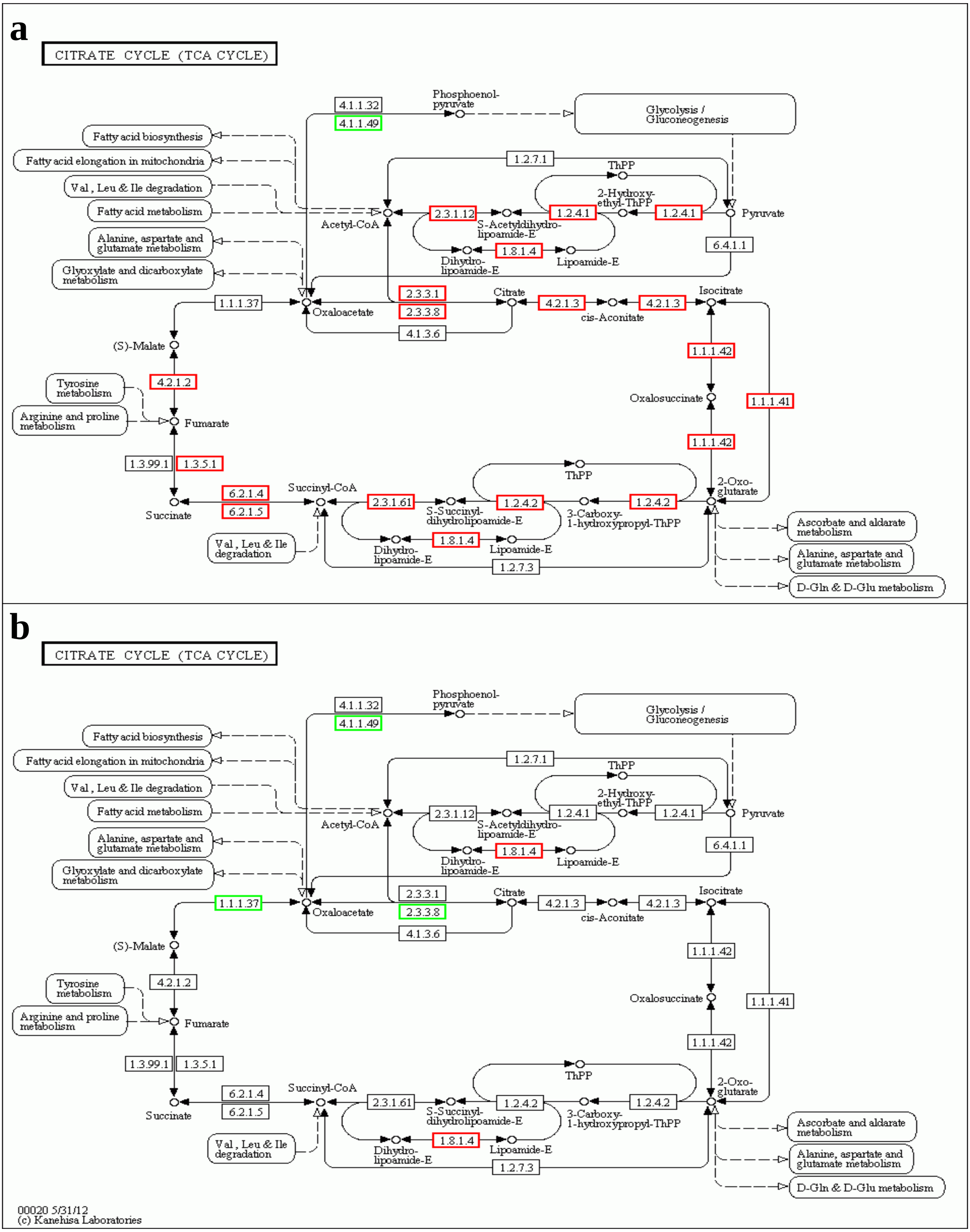

Supplementary Material 3 KEGG pathway visualization of Citrate cycle (TCA cycle)
a: A-VS-B; b: D-VS-E. KEGG pathway analysis of significant differentially expressed transcripts in Citrate cycle (TCA cycle) in Tibetan hulless barley under detached water-deficit stress. In the figure, up-regulated genes are marked with red borders and down-regulated genes with green borders. Non-change genes are marked with black borders.
