# Supplementary material for: Dehydration induced transcriptomic responses in two Tibetan hulless barley (Hordeum vulgare var. nudum) accessions distinguished by drought tolerance
Source: BMC Genomics. 2017 Oct 11;18:775. doi: 10.1186/s12864-017-4152-1 (PMC5637072; doi:10.1186/s12864-017-4152-1)
Supplement: Supplementary file 4 — KEGG pathway visualization of Wax biosynthesis. (PDF 95 kb) [file 12864_2017_4152_MOESM4_ESM.pdf]

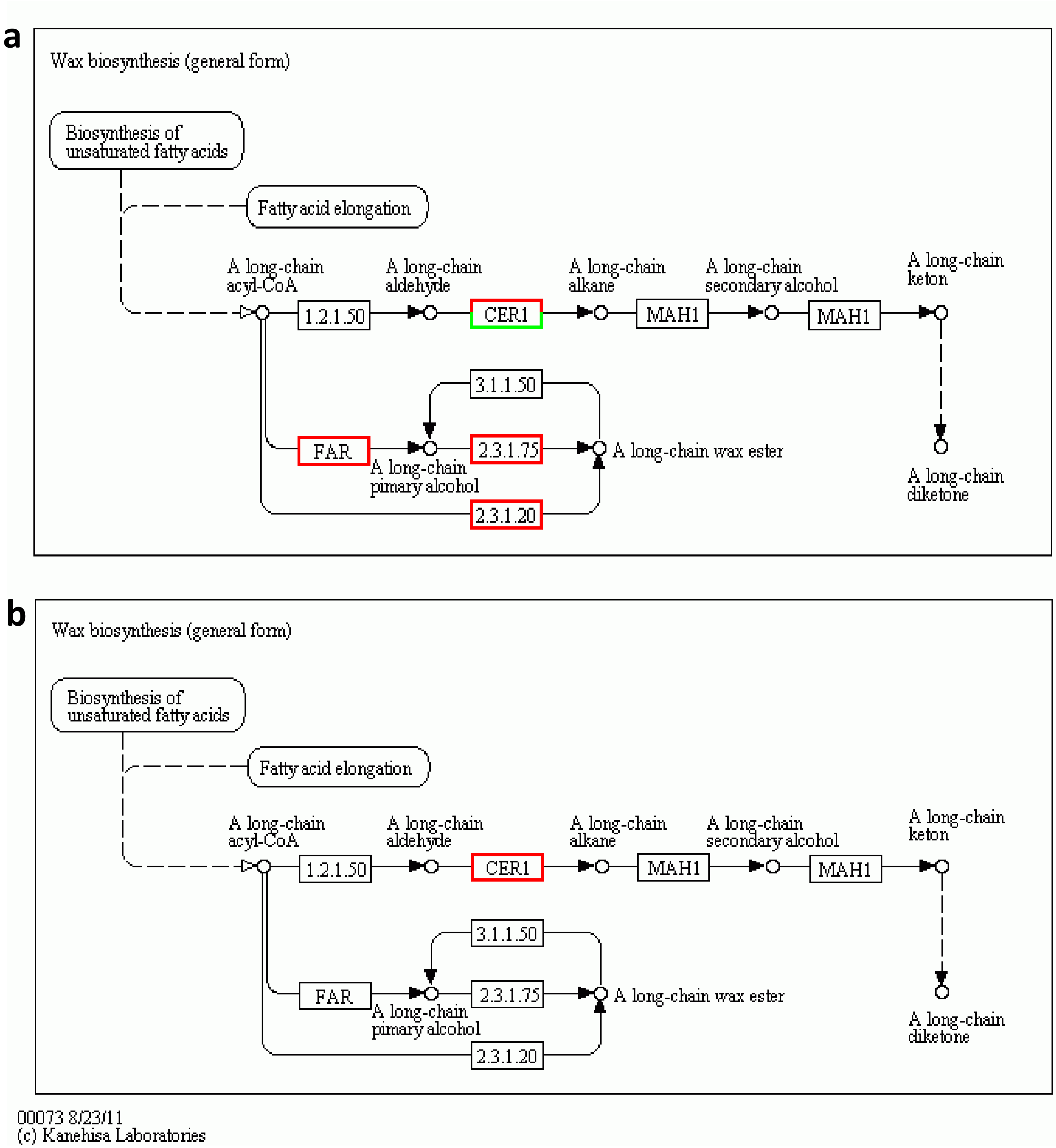

Supplementary Material 4 KEGG pathway visualization of Wax biosynthesis

a: A-VS-B; b: D-VS-E. KEGG pathway analysis of significant differentially expressed transcripts in Wax biosynthesis in Tibetan hulless barley under detached water-deficit stress. In the figure, up-regulated genes are marked with red borders and down-regulated genes with green borders. Non-change genes are marked with black borders.
