# Supplementary material for: Dehydration induced transcriptomic responses in two Tibetan hulless barley (Hordeum vulgare var. nudum) accessions distinguished by drought tolerance
Source: BMC Genomics. 2017 Oct 11;18:775. doi: 10.1186/s12864-017-4152-1 (PMC5637072; doi:10.1186/s12864-017-4152-1)
Supplement: Supplementary file 10 — Candidate genes to enhance drought tolerance. (DOCX 29 kb) [file 12864_2017_4152_MOESM10_ESM.docx]

**Candidate genes to enhance drought tolerance**

Firstly, we focused on genes which showed a continued up-regulated pattern in both Z772 and Z013. These genes included auxin-repressed protein (HVU026276.1), which may function as a repressor of plant growth and an activator of disease resistance [1]; asparagine synthetase (HVU008925.1); dehydrins (HVU005858.3 and HVU035383.1); ferritin (HVU026815.1), which serves in iron storage (in a non-toxic form) and transportation; Na^+^/H^+^ antiporter (HVU013527.1), a membrane pump which is responsible for maintaining the balance of pH, HVU013527.1 is also similar to *WALI7*, an aluminum induced genes in wheat roots [2]; ribonucleoprotein complex (HVU017792.1), which is similar to a ribosome-binding factor PSRP1 (XM_003559596.2, *Brachypodium distachyon*); homeobox-leucine zipper protein (HVU006047.1), a class of eukaryotic transcription factors; protein phosphatase 2C (HVU014205.1 and HVU020466.1); cold regulated protein (HVU005148.1); UDP-D-glucose epimerase (HVU031245.1), which works in galactose metabolism, acts on carbohydrates and derivatives; protease inhibitor (HVU023754.1), very similar to H. *vulgare* *Bsi1* gene, which may have potential roles in defense against fungal attack [3].

Secondly, we focused on DEGs which also showed a continued up-regulated pattern during dehydration stress but the expression was significant higher in Z772 than in Z013 at least at 1 h. These genes include F-box/kelch-repeat protein (HVU026571.1), protein containing at least one F-box domain and kelch repeat, may associate with cellular functions such as signal transduction and regulation of the cell cycle; Malate-CoA ligase (HVU036172.2), which functions in the polymerization pathway of polymalic acid; cathepsin A (carboxypeptidase C, HVU002653.1), an acidic serine carboxypeptidase, which can regulate chaperone-mediated autophagy through cleavage of the lysosomal receptor, is an multifunctional enzyme with distinct protective and catalytic function [4]; cytochrome P450 (HVU007840.1), the terminal oxidase enzymes in electron transfer chains; calcium-binding protein CML (HVU023104.1), proteins that participate in calcium cell signaling pathways by binding to Ca^2+^; wax-ester synthase/diacylglycerol O-acyltransferase (HVU012911.1), a key enzyme in the synthesize of plant wax ester; protein phosphatase 2C (HVU013443.3); cystinosin homolog (HVU013720.1), which has a seven-transmembrane (TM) domain structure and may be location to lysosomal; phosphatidylglycerol/phosphatidylinositol transfer protein (HVU020126.1), is an ubiquitous cytosolic domain involved in transport of phospholipids from their site of synthesis in the endoplasmic reticulum and Golgi to other cell membranes; beta-glucosidase (HVU011108.1), an enzyme which can hydrolyze phytohormone glucosyl conjugates to release active phytohormones, to regulate cellular activities [5].

We also focused on those DEGs, which were highly up-regulated at 1 h but down-regulated or unchanged at 5 h. These genes included spermidine synthase (HVU002314.1), which participate in the biosynthesis of spermidine, a precursor to polyamines, such as spermine and thermospermine, most of which contribute to tolerance against drought and salinity in plants; nudix hydrolase 8 (HVU000639.1), nudix hydrolases have been shown to catalyze the hydrolysis of nucleoside diphosphates and pyridine nucleotides, it was reported that AtNUDX8 appears in the fibroblast growth factor type Nudix enzyme (FGFTNE) subfamily in a monophyletic clade, which have hydrolase activity towards ADP-ribose and NADH, is important for defense responses in plants [6]; chaperone protein dnaJ (HVU007764.1), also known as Hsp40, is a molecular chaperone protein expressed in a wide variety of organisms from bacteria to humans, protect proteins from irreversible aggregation during synthesis and in times of cellular stress; polyamine oxidase (HVU031846.1), one of the key enzymes which catalyze the degradation of polyamine, is very important for the regulation of polyamine concentration in plant cells, it was reported that it functions in cell wall hardening, programmed cell death and cellular solute antioxidant protection system; AP2/EREBP-like transcription factor (HVU007121.1 and HVU030870.1), which plays a critical role in plant development and in adaptation to abiotic stress conditions [7]; nitrate transporter (HVU038692.1), functions in the uptake and redistribution of NO_3_^-^ within plant, which belongs to a large gene family, may also has other functions such as absorption of other elements and improving the resistance to biotic and abiotic stresses; heat shock protein Hsp20 (HVU036883.1 and HVU014948.1), act as protein chaperones that can protect plants by preventing protein aggregation during biotic and abiotic stress conditions [8]. It was proposed a Hsp17.8 in Arabidopsis functions as an AKR2A cofactor in targeting membrane proteins to plastid outer membranes under normal physiological conditions [9] and it was reported that its over-expression in transgenic lettuce can give rise to dehydration and salt stress resistance [10]; S-like Rnase, an important family of RNA-degrading enzymes in plants, are close molecular relatives to the S-Rnases, but their functions are not restricted to pistil but also in phosphate-starvation processes, senescence, wounding and pathogen defense [11], it was reported that the over-expression of an S-like ribonuclease gene, OsRNS4, confers enhanced tolerance to high salinity in rice [12]; F-box proteins (HVU006270.1), are proteins containing at least one F-box domain, which functions in signal transduction and regulation of the cell cycle; jasmonate ZIM domain-containing protein (HVU013546.1), which is a key mediators of jasmonate signalling include MYC transcription factors [13]; zinc finger protein CONSTANS-like (HVU000680.1), a B-box-type zinc finger domain containing protein, is a transcription activator and may be involved in fruit ripening and stress responses [14]; subtilisin-like proteases (HVU010846.1), also named subtilases, are serine proteases that fulfill highly specific functions in plant development and signaling cascades, several subtilisin-like proteases are associated to plant–pathogen resistance [15]; SNF1-type serine-threonine protein kinase (HVU018682.1), which was studied extensively by many researches, play essential roles in regulating stress-responsive gene expression in response to many biotic and abiotic stress; protein phosphatase 2C (HVU037966.1 and HVU021931.1); hyperosmolality-gated Ca^2+^ permeable channel (HVU011974.1), voltage-gated ion channels with a permeability to the calcium ion Ca^2+^, which serves as an important second messenger participating in signal transduction of various environmental stresses; Glucan 1,3-beta-Glucosidase (HVU014782.1), an exocellulase with specificity for 1,3-beta-D-glucasidic linkages, catalyzes hydrolysis of beta-D-glucose units from the non-reducing ends of 1,3-beta-D-glucans, releasing glucose.

**References**

1. Zhao Y, Li C, Ge J, Xu M, Zhu Q, Wu T, Guo A, Xie J, Dong H. Recessive mutation identifies auxin-repressed protein ARP1, which regulates growth and disease resistance in tobacco. *Mol Plant Microbe Interact*. 2014;27(7):638–654.
2. Richards KD, Snowden KC, Gardner RC. *Wali6* and *wali7*. Genes induced by aluminum in wheat (*Triticum aestivum* L.) roots. *Plant Physiol*. 1994;105(4):1455–1456.
3. Stevens C, Titarenko E, Hargreaves JA, Gurr SJ. Defence-related gene activation during an incompatible interaction between *Stagonospora* (*Septoria*) *nodorum* and barley (*Hordeum vulgare* L.) coleoptile cells. *Plant Mol Biol*. 1996;31(4):741–749.
4. Timur ZK, Akyildiz Demir S, Seyrantepe V. Lysosomal Cathepsin A Plays a Significant Role in the Processing of Endogenous Bioactive Peptides. *Front Mol Biosci.* 2016;3:68.
5. Hua YL, Ekkhara W, Sansenya S, Srisomsap C, Roytrakul S, Saburi W, Takeda R, Matsuura H, Mori H, Ketudat Cairns JR. Identification of rice Os4BGlu13 as a β-glucosidase which hydrolyzes gibberellin A4 1-O-β-D-glucosyl ester, in addition to tuberonic acid glucoside and salicylic acid derivative glucosides. *Arch Biochem Biophys*. 2015;583:36–46.
6. Gunawardana D, Likic V, Gayler KR. A comprehensive bioinformatics analysis of the Nudix superfamily in *Arabidopsis thaliana*. *Comp Funct Genomics.* 2009:820381.
7. Pandey B, Sharma P, Tyagi C, Goyal S, Grover A, Sharma I. Structural modeling and molecular simulation analysis of HvAP2/EREBP from barley. *J Biomol Struct Dyn*. 2015;22:1–44.
8. Muthusamy SK, Dalal M, Chinnusamy V, Bansal KC. Genome-wide identification and analysis of biotic and abiotic stress regulation of small heat shock protein (*HSP20*) family genes in breadwheat. *J Plant Physiol*. 2017;211:100–113.
9. Kim DH, Xu ZY, Na YJ, Yoo YJ, Lee J, Sohn EJ, Hwang I. Small heat shock protein Hsp17.8 functions as an AKR2A cofactor in the targeting of chloroplast outer membrane proteins in Arabidopsis. *Plant Physiol*. 2011;157(1):132–146.
10. Kim DH, Xu ZY, Hwang I. *AtHSP17.8* overexpression in transgenic lettuce gives rise to dehydration and salt stress resistance phenotypes through modulation of ABA-mediated signaling. *Plant Cell Rep*. 2013;32(12):1953–1963.
11. Liang L, Lai Z, Ma W, Zhang Y, Xue Y. *AhSL28*, a senescence- and phosphate starvation-induced S-like RNase gene in *Antirrhinum*. *Biochim Biophys Acta*. 2002;1579(1):64–71.
12. Zheng J, Wang Y, He Y, Zhou J, Li Y, Liu Q, Xie X. Overexpression of an S-like ribonuclease gene, *OsRNS4*, confers enhanced tolerance to high salinity and hyposensitivity to phytochrome-mediated light signals in rice. *Plantsci*. 2014;214:99–105.
13. Zhang F, Yao J, Ke J, Zhang L, Lam VQ, et al. Structural basis of JAZ repression of MYC transcription factors in jasmonate signalling. *Nature.* 2015;525(7568):269–273.
14. Chen J, Chen JY, Wang JN, Kuang JF, Shan W, Lu WJ. Molecular characterization and expression profiles of *MaCOL1*, a *CONSTANS*-like gene in banana fruit. *Gene*. 2012;496(2):110–117.
15. Figueiredo A, Monteiro F, Sebastiana M. Subtilisin-like proteases in plant-pathogen recognition and immune priming: a perspective. *Front Plant Sci*. 2014;5:739.
